# Multipass CCS Refiner: A Web Application for Accurate Collision Cross Section Calibration in Cyclic Ion Mobility-Mass Spectrometry

**DOI:** 10.1021/jasms.5c00199

**Published:** 2025-08-19

**Authors:** Eric C. Gier, Dmitry Leontyev, Facundo M. Fernández

**Affiliations:** † School of Chemistry and Biochemistry, 1372 Georgia Institute of Technology, Atlanta, Georgia 30332, United States; ‡ Parker H. Petit Institute for Bioengineering and Bioscience, Atlanta, Georgia 30332, United States

**Keywords:** multipass CCS Refiner, collision cross section calibration, cyclic ion mobility spectrometry, traveling-wave perturbation-correction

## Abstract

In this paper, we present the *Multipass CCS Refiner*, an R application for calibrating multipass collision cross section (CCS) measurements with cyclic ion mobility spectrometry (cIMS). The *Multipass CCS Refiner* combines the necessary tools for calculating accurate CCS measurements from multipass cIMS experiments into a user-friendly web-based Shiny interface accessible regardless of programming knowledge. The application provides a suite of functions for calculating accurate arrival times, automated pass counting approximation, correction of arrival time perturbations during separation, constructing calibration curves unique to specific instrument settings, separating fine mobility features, and a variety of visualization tools. Code for the application is structured to be easily modified to meet the user’s needs. The *Multipass CCS Refiner* implements the calibration approach described by Lin and Costello to reduce the need for carefully selected separation times while accounting for artifacts underlying fluctuations in measured ion arrival times. The application is showcased with downloadable data files of commonly used standards, which can be run in the app directly or edited with user experimental data.

## Graphical abstract

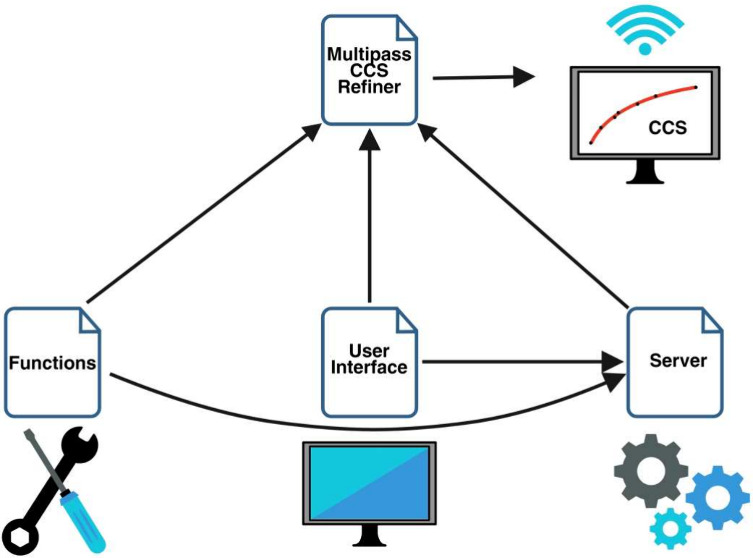

## Introduction

Ion mobility spectrometry (IMS) is a powerful analytical technique for separating gas-phase ions based on their collision frequency and intensity with a buffer gas. Drift tube IMS (DTIMS) instruments employ uniform electric fields to derive ion collision cross sections (CCS) from first-principles; however, their resolving power is constrained by practical factors such as the ion path length.^1^ In response to the demand for improved separations, a variety of IMS platforms have been developed, achieving mobility resolving powers in the hundreds.^2,3^ One such platform is cyclic IMS (cIMS) often coupled to mass spectrometry (cIMS-MS), in which ions traverse a 1-m closed-loop multiple times under the influence of a traveling wave (TW) electric field.^4^ Unlike DTIMS, cIMS does not permit direct calculation of CCS values under the nonstatic electric field conditions employed. Instead, users typically employ calibration strategies with a known standard. Mobility calibration requires either single pass experiments or the generation of multiple calibration curves using carefully selected separation times to ensure that analytes and calibrants complete an equivalent number of passes.

Accurate multipass CCS measurements—within 1% error of reference DTIMS values—can be obtained by evaluating the average ion velocity across different path lengths.^5^ However, this method does not fully account for the underlying physical principles responsible for variations in measured arrival times at different separation time points.^6,7^ An alternative calibration strategy proposed by Lin and Costello addresses this issue by correcting for arrival time perturbations introduced by electric field switching when ions are transferred into the time-of-flight detector.^7^ This approach uses a linear relationship based on the ion path length to calculate a perturbation-corrected multipass drift time from measurable parameters. Implementation of this method, however, requires measurements at multiple separation times, which—when performed manually—can considerably increase postprocessing effort. Previous studies have also demonstrated the effectiveness of CCS calibration strategies in extended path length IMS platforms, such as structures for lossless ion manipulation, supporting their utility for high-resolution ion mobility measurements.^8,9^

At present, no widely accessible tools exist to support the multipass calibration of cIMS-MS systems, thereby hindering the broader adoption of refined multipass CCS strategies. To address this limitation, we introduce the *Multipass CCS Refiner*, a custom R script equipped with a Shiny graphical user interface. This tool enables users to perform multipass CCS calibration following the Lin and Costello approach, regardless of programming expertise. The *Multipass CCS Refiner* streamlines the process of generating calibration curves and calculating CCS values from multipass data at user selected separation times. Calibration curves can be tailored to specific experimental conditions using user-supplied reference standards with known CCS values, allowing the use of calibrants best suited for the molecular class or ion type under investigation.^10^ Additionally, the application allows users to download example and result files to facilitate data uploading, processing, and visualization.

## Methods

### Materials

The Major Mix calibration standard was obtained from Waters Inc. (Wilmslow, U.K.). The SPLASH II Lipidomix standard was purchased from Sigma-Aldrich Inc. (St. Louis, MO, USA). Prostaglandin isomers A2 and J2 were obtained from Cayman Chemical (Ann Arbor, MI, USA).

### Code and Application Details

The *Multipass CCS Refiner* is hosted online via Posit and can be accessed at: https://ericgier.shinyapps.io/Multipass-CCS-Refiner/. Complete documentation is available on GitHub: https://github.com/facundof2016/Multipass-CCS-Refiner. The application was developed in the R programming language (version 4.5.0), using RStudio (version 2025.05.1), and leverages the R Shiny framework (version 1.10.0) to provide an interactive web-based interface.^11^ The following R packages are required to run the application locally: shiny, shinythemes, bslib, shinyjs, shinyBS, DT, readxl, xlsx, ggplot2, grid, and viridisLite. Importantly, none of these packages or environments are required for online use. A detailed breakdown of major code components is provided in the Supporting Information (Table S1).

The application is structured into four core modules: (1) a functional module for data processing, plotting, and CCS calculations; (2) a user interface module for data input; (3) a server module that integrates user input with backend operations; and (4) a parent module that combines all elements for real-time interactive execution via the web. Data upload functionality is optimized for compatibility with MassLynx output files (Waters Inc., Wilmslow, U.K.). Arrival time distribution (ATD) data should be first compiled into.xlsx files; editable formatting templates can be downloaded from either the “Using the Application” section within the app’s Introduction tab or directly from GitHub. For streamlined data analysis, users are encouraged to download and modify the example files. A video tutorial is also available on the Fernández Group YouTube channel: www.youtube.com/watch?v=AXrbocbQGY8&t=0s.

### Ion Mobility–Mass Spectrometry

All experiments were performed using a SELECT SERIES Cyclic IMS mass spectrometer (Waters Inc., Wilmslow, U.K.), operated in positive ion mode with direct infusion electrospray ionization. Single-pass and multipass periodic drift time calibration curves were generated using the *Multipass CCS Refiner*, with calibration based on Major Mix across the mass range of *m*/*z* 311–1013, using literature-reported CCS values.^12^ Multipass separation times were selected to ensure a minimum of four passes per calibrant, and the number of passes for each analyte was determined by using the automated pass counter within the *Multipass CCS Refiner*. Spectra exhibiting wrap-around effects were excluded from analysis, and a minimum of six data points per calibrant were used to calculate perturbation-corrected periodic drift times according to the Lin and Costello method. Detailed experimental parameters are available in the Supporting Information (Table S2), along with an illustrative example of the Lin and Costello calibration approach (Figure S1).

CCS values for standards in the SPLASH II Lipidomix mixture were calculated using both the *Multipass CCS Refiner* and the Waters single-pass CCS calibration spreadsheet, a tool commonly employed in cIMS-MS experiments. Prostaglandin isomers A2 and J2 were used to demonstrate the application’s capacity to resolve multiple peak arrival time distributions.^13^ All measurements were performed by using identical instrument settings and were collected sequentially on the same day to ensure consistency with calibration conditions.

## Results and Discussion

The *Multipass CCS Refiner* automates the calculation of perturbation-corrected periodic drift times (t_pp_) using the method described by Lin and Costello, thereby enabling accurate multipass CCS determinations under user-defined separation conditions.^7^ For each calibrant, the precise arrival time (t_n_) is determined by fitting a Gaussian function to the raw ATD using nonlinear least-squares regression (Figure 1A).^14^ Linear models used to calculate t_pp_ are constructed from data acquired at multiple separation times (t_s_). Each data point in the model is defined by the number of passes through the cyclic separation cell (n), the applied separation time (t_s_), and the total drift time (t_nd_), where t_nd_ is calculated as the difference between the measured t_n_ and the bypass time determined at t_s_ = 0.01 ms (Figure 1B).

**Figure 1:**
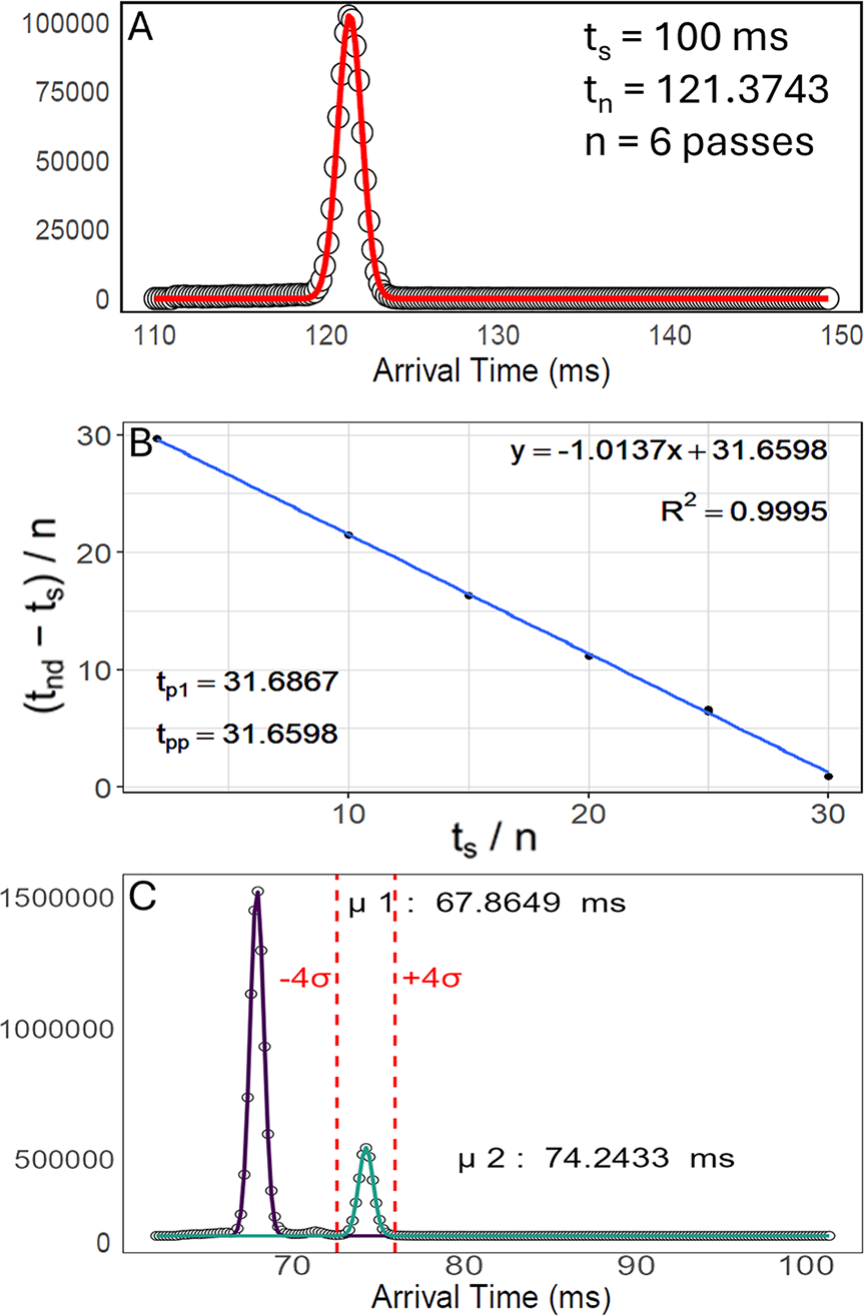
(A) ATD of the [M + H]^+^ ion of leucine enkephalin acquired at a t_s_ of 100 ms, with the Gaussian fit overlaid in red. (B) Linear model constructed from data for the polyalanine ion at *m*/*z* 942. (C) ATDs of prostaglandins J2 and A2 with corresponding Gaussian fits shown in purple and green, respectively.

The *Multipass CCS Refiner* estimates the number of passes completed at each t_s_ using an automated pass counter, which leverages single-pass (t_p1_) and bypass measurements. For nonisomeric species, the application facilitates calibration by providing arrival time calculations based on the most intense peak in the ATD. While smaller, resolved mobility features can still be isolated using manual analysis or slicing experiments, the application offers a rapid and user-friendly alternative for processing and visualization (Figure 1C).

Calibration curves are generated within the application by compiling t_p1_ and t_pp_ values across all calibrants and fitting the data to a power law function (Figure 2). The Lin and Costello correction method is then applied to experimental analytes and uses these calibration curves to compute multipass CCS values. The capabilities of the *Multipass CCS Refiner* are demonstrated through the construction of calibration curves using the Waters Major Mix standard, calculation of experimental CCS values for standards in the SPLASH II Lipidomix mixture, and resolution of multiple-peak ATDs for prostaglandin isomers A2 and J2. Linear models for calibrants in Waters Major Mix (Figure S2), SPLASH II Lipidomix (Figure S3), and prostaglandin isomers (Figure S4) can be reproduced by using the corresponding example files included within the application.

**Figure 2:**
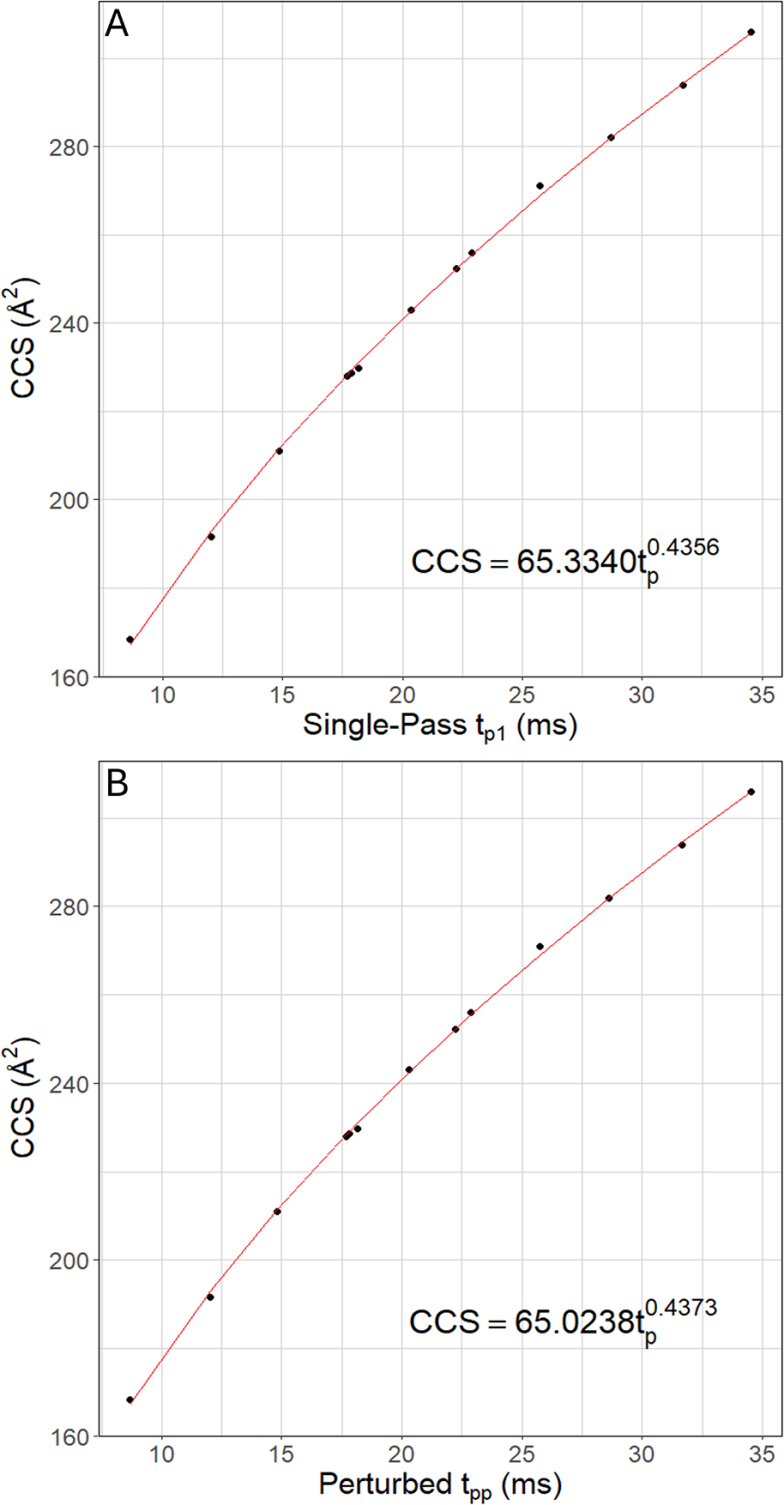
Calibration curves generated by the *Multipass CCS Refiner* for Waters Major Mix calibrants using power law fits based on (A) single-pass arrival time measurements (t_p1_) and (B) perturbation-corrected periodic drift times (t_pp_).

The application’s graphical user interface (GUI) is organized into a series of tabbed panels designed for intuitive navigation. Upon launching the application, users are directed to the *Introduction* tab, which provides background information, usage instructions, downloadable calibration and experimental input files, and relevant references. The *Create Calibration Curves* tab enables the generation of calibration curves from comma-separated lists containing analyte names and corresponding CCS values. Users may optionally input experimental parameters, save calibration results, and visualize the resulting calibration curves displayed at the bottom of the interface. Once a calibration curve has been saved, the *Process Data* tab can be used to calculate CCS values for experimental analytes with the option to compare them against known or predicted CCS values. Previously saved calibration curves can be accessed via the *Calibration Curve* panels. Results generated in the *Process Data* tab are presented as an interactive data frame, and plots corresponding to individual or all analytes can be downloaded in.pdf format. When running the application locally in RStudio, all plots are displayed in the *Plots* pane, allowing for convenient export to additional file formats. The application also supports the reconstruction of ATDs from unresolved mobility features and those exhibiting peak-splitting due to wrap-around effects, as previously described.^7^ Reconstructed ATDs can be incorporated into the workflow to calculate perturbation-corrected multipass CCS values by using the Lin and Costello method.

To evaluate the accuracy of the *Multipass CCS Refiner*, CCS values for standards in the SPLASH II Lipidomix mixture were calculated using both single-pass and multipass workflows and compared to values generated using the Waters single-pass CCS calibration spreadsheet under identical instrument conditions (Table 1). Single-pass and multipass CCS values obtained from the *Multipass CCS Refiner* were consistent within 0.2% of each other and exhibited agreement with 1.5% of the values produced by the Waters workflow.

**Table 1:** Comparison of CCS Values Obtained Using the Waters Single-Pass CCS Calibration Workflow with Both Single-Pass and Multipass Measurements Generated by the *Multipass CCS Refiner*, with Percent Differences Representing the Deviation of *Multipass CCS Refiner* CCS Values from Those Calculated by Using the Waters Workflow

Standard	Adduct	^cIMS^CCS_N2_ Single-pass Instrument Default	^cIMS^CCS_N2_ Single-pass CCS Refiner	% Difference Single-pass	^cIMS^CCS_N2_ Multipass CCS Refiner	% Difference Multipass
d7-PC (15:0–18:1)	[M + H]^+^	292.8	289.1	1.28	288.6	1.44
d7-LysoPC (18:1)	[M + H]^+^	236.0	238.9	1.22	238.8	1.19
d7-PE (15:0–18:1)	[M + H]^+^	280.1	279.2	0.32	279.2	0.32
d7-LysoPE (18:1)	[M + H]^+^	218.9	222.1	1.45	222.2	1.50
d7-LysoPE (18:1)	[M + Na]^+^	225.1	228.2	1.38	228.2	1.37
d7-LysoPC (18:1)	[M + Na]^+^	238.6	241.4	1.18	241.3	1.13

To further test the potential of this application, the *Multipass CCS Refiner* was employed to calculate perturbation-corrected CCS values for differential features detected in human renal cell carcinoma liver tissue samples.^15^ For eight lipid species with annotations confirmed by liquid chromatography-tandem mass spectrometry (LC-MS/MS),^16^ CCS measurements achieved an average deviation of just 0.4% relative to reference database values. Importantly, the application also enabled the characterization of previously unidentified differential features in the absence of direct LC-MS/MS validation, underscoring the broader utility of multipass CCS refinement in untargeted discovery workflows. Although the current implementation has been primarily applied to lipid analyses, the underlying principles of traveling wave perturbations to ion velocity in cIMS platforms are broadly applicable and may extend to other molecular classes.

## Conclusions

The *Multipass CCS Refiner* provides an accessible and efficient platform for calibrating multipass CCS measurements acquired on cIMS-MS instruments. The application allows users to upload data, generate calibration curves, and visualize results with minimal manual preprocessing. Its user-friendly interface, built-in data formatting tools, and automated calculation pipeline significantly reduce both the time and computational expertise required to implement multipass CCS workflows. Researchers are encouraged to download the full version of the code from GitHub and customize it to meet the specific needs of their experimental workflows.

## Supplementary Material



## Notes

Description of the *Multipass CCS Refiner* module organization; instrument parameters; summary of the Lin and Costello method illustrated with the protonated adduct of Val-Tyr-Val; (*t_nd_* – *t_s_*)/*n* versus *t_s_*/*n* plots of major mix calibrants; (*t_nd_* – *t_s_*)/*n* versus *t_s_*/*n* plots of SPLASH II Lipidomix standards; ATDs of the sodium adducts of prostaglandins A2 and J2 and their (*t_nd_* – *t_s_*)/*n* versus *t_s_*/*n* plots (PDF)
