# Supplementary material for: Multipass CCS Refiner: A Web Application for Accurate Collision Cross Section Calibration in Cyclic Ion Mobility-Mass Spectrometry
Source: J Am Soc Mass Spectrom. 2025 Aug 19;36(9):2000–4. doi: 10.1021/jasms.5c00199 (PMC12412177; doi:10.1021/jasms.5c00199)
Supplement: Supplementary file 1 [file js5c00199_si_001.pdf]

Supporting Information

Multipass CCS Refiner: A Web Application for Accurate Collision Cross Section Calibration in Cyclic Ion Mobility-Mass Spectrometry.

Eric C. Gier[1], Dmitry Leontyev[1], Facundo M. Fernández[1,2*]

[1]School of Chemistry and Biochemistry, Georgia Institute of Technology, Atlanta, GA 30332 (USA).

[2]Parker H. Petit Institute for Bioengineering and Bioscience, Atlanta, GA 30332 (USA).

*Email: facundo.fernandez@chemistry.gatech.edu

Contents:

Table S1. Overview of Multipass CCS Refiner Code Structure and Functions.

Table S2. Instrument Parameters for cIMS Data Collection.

Figure S1. Example Calculation of Perturbation Corrected Periodic Drift Time.

Figure S2. Linear Fits for Major Mix Standards.

Figure S3. Linear Fits for SPLASH II Lipidomix Standards.

Figure S4. Multiple Peak Arrival Time Distribution Separation of Prostaglandin Isomers.

Table S1. Multipass CCS Refiner Code Organization and Major Functionalities.

Module	Category	Name	Description
Functions	Calculation	calculate_arrival_time	Calculates precise arrival times from raw ATDs using nonlinear least-squares regression from guess values near the maximum intensity. Called by the process_sheet function and returns a fitted model.
		calculate_ccs_with and without_comparison	Functions for calculating CCS values from single pass and perturbed periodic drift times. The with_comparison function compares CCS values to values specified by the user. Returns a lists of calculated CCS values, percent differences and plot objects. Called by the input$runData observer.
		calculate_multiple_arrival_times	Calculates precise arrival times from raw ATDs using nonlinear least squares regression for up to 10 peaks. Accepts input guess values specified by the user and returns plot objects. Called by input$generate observer.
	Plotting	plot_gaussian_arrival	Plots a fitted Gaussian model overtop raw data from ATDs. Returns model coefficients and a plot object. Called by the process_sheet function.
		plot_linear_chart	Creates a linear plot from precise arrival time data and returns model coefficients and a plot object. Called by the process_sheet function.
		plot_multiple_gaussians	Plots fitted gaussian distributions overtop raw data from user supplied guess values. Called by the input$generate observer.
		plot_power_curve	Plots calibration power curves and returns the power curve equation. Called by the input$runCalibration observer.
	Processing	process_sheet	Reads a sheet from a .xlsx file containing all ATDs for a single analyte. Calls calculate_arrival_time and plot_linear_chart. Conducts automated pass counting using a rounded linear relationship. Returns lists of arrival times and plots. Called by the input$runCalibration and input$runData observers.
Interface	Interface tab panel	Introduction	Contains dropdown menus for background information, using the application and citations. Triggers the

			output$exampleCalibrationFile, output$exampleExperimentFile and output$exampleMultiPeakATDFile download handlers for example data.
		Create Calibration Curves	Accepts user input for calibration curve construction and triggers the input$runCalibration, input$save, output$downloadAllCalibrationPlots and output$downloadCalibrationCurves observers and download handlers. Contains single and multiple pass calibration curves at the bottom of the tab.
		Process Data	Accepts user input for processing data and triggers the input$runData and output$downloadAllDataPlots observer and download handler.
		Multiple Peak ATD	Accepts and plots ATDs based on user input of data location.
		Calibration Curves 1 and 2	Displays saved calibration curves and settings for the user to review within the application. Triggered by the input$save observer. Triggers the input$delete observer.
		Settings	Location for decimal place settings for plots and CCS percent difference calculations.
Server	Observer	input$save	Saves calibration curves to calibration curve tabs and is triggered from the Create Calibration Curves tab. Saving a calibration curve is required to process data from a calibration curve slot.
		input$delete	Deletes saved calibration curve data from calibration curve slots and is triggered from the Calibration Curve tabs.
		input$runCalibration	Triggerable after user inputs for analyte names, CCS values and filepath are entered in the Create Calibration Curve tab. Calls the process_sheet and plot_power_curve functions after all error checks pass. Increments a progress bar after each sheet is processed.
		input$runData	Triggerable after user inputs for analyte names, CCS values and filepath are entered in the Process Data tab. Calls the process_sheet and calculate_ccs functions after all error checks pass. Increments a progress bar after each sheet is processed. Outputs a summary

			of calculations in a dataframe to the Process Data tab.
		input$plotRawData	Outputs plots of raw ATDs to the Multiple Peak ATD tab for users to make educated guess values for peaks within an ATD.
		input$generate	Calls calculate_multiple_arrival_times and plot_multiple_gaussians functions to render plots of fitted ATDs to the Multiple Peak ATD tab.
		input$peakSelection, input$sigmaRange	Updates plots created from input$generate in the Multiple Peak ATD tab.
		Input$copytoClipboard	Combines raw data from within a user-specified range with generated intensities from a fitted model. Results of the reconstructed ATD are copied to the clipboard.
	Download Handler	output$exampleCalibrationFile, output$exampleExperimentFile and output$exampleMultiPeakATDFile	Allows the user to download examples from the Introduction tab as .xlsx files.
		output$downloadAllCalibrationPlots	Allows the user to download calibration curves, fitted ATDs and linear plots for calculating perturbed periodic drift time for calibration data in .pdf format from the Create Calibration Curve tab.
		output$downloadCalibrationCurves	Allows the user to download calibration curves for calibration data in .pdf format from the Create Calibration Curve tab.
		output$downloadAllDataPlots	Allows the user to download fitted ATDs and linear plots for calculating perturbed periodic drift time for user data in .pdf format from the Process Data tab.

Table S2. Summary of Instrument Settings for cIMS Data Acquisition.

Global Settings	
Instrument Polarity	Positive
Analyzer Mode	V-mode
Infusion Flow Rate (μL/min)	2
Mass Range (*m/z*)	0-1200

Electrospray Ionization	
Capillary (kV)	3
Cone (V)	20
Source Offset (V)	10
Source Temperature (°C)	100
Desolvation Temperature (°C)	250
Cone Gas (L/hour)	0
Desolvation Gas (L/hour)	800
Nebuliser Gas (Bar)	6
Reference Capillary (kV)	3

StepWave	
Body Gradient (V)	20
Head Gradient (V)	10
Ion Guide 1 Offset (V)	3
Ion Guide 2 Offset (V)	0.3
Diff Ap 2 (-V)	0
IG TW Velocity (m/s)	150
IG TW Pulse Height (V)	0.4

Cyclic Control Page	
ADC Start Delay	Automatic
Pushes Per Bin	3
Number of Bins	200
Cyclic TW Velocity (m/s)	375
Array TW Velocity (m/s)	375
TW Static Height (V)	18
Separation time (ms)	Varied*

Quad/MS Profile/DRE	
MS Mode	MS
MS Profile	Automatic
Ion Energy (V)	0.4
Pre-filter (V)	2
Trap CE (V)	6
Transfer CE (V)	4
Detector Voltage (V)	2058
DRE Lens	Off

Trap	
Trap TW Velocity (m/s)	311
Trap TW Pulse Height (V)	4
Trap Entrance (V)	2
Trap Bias (V)	2
Trap DC (V)	-4
Trap Exit (V)	0
Post Trap Gradient (V)	3
Post Trap Bias (V)	35
Collision Gas Setting	Gas 1 to Trap/Transfer
Collision Gas 1 (ml/min)	3.5

Cyclic IMS	
Helium Entrance (V)	10
Helium Cell Bias (V)	30
Helium Exit (-V)	20
Pre IMS Reference (V)	85
Racetrack Bias (V)	70
Repeller (V)	100

Transfer	
Pre Transfer Gradient (V)	7
Pre Transfer Bias (V)	0
Transfer Entrance (V)	2
Transfer Gradient (V)	4
Transfer Exit (-V)	15

RF	
StepWave RF (V)	200
Ion Guide RF (V)	300
Trap RF (V)	400
Drift cell RF (V)	300
Pre/Post Array RF (V)	350
Cyclic RF (V)	250
Transfer RF (V)	500
Transfer RF Gain	5

*For all experiments a by-pass spectrum was collected with a separation time of 0.01 ms; a single-pass spectrum, at 2 ms; and multipass spectra were collected at 10, 15, 30, 32, 50, 75, and 100 ms.

Summary of the Lin and Costello Calibration Method:

The multipass CCS calibration method developed by Lin and Costello accounts for arrival time perturbations introduced by electric field switching at the separation time (t_s), when ions are permitted to enter the time-of-flight detector. The authors show that a linear relationship based on ion path length can be employed to calculate a perturbation-corrected multipass drift time (t_{pp}) from experimentally measurable variables (Equation 1). These variables include the total drift time prior to ion exit from the mobility cell (t_{nd}), the number of passes through the cyclic ion mobility separation cell (n), and the separation time (t_s). In this model, the slope reflects the ratio of ion velocities in the unperturbed (v_u) and perturbed (v_p) states, corresponding to ion motion before and after the onset of t_s, respectively.

$$\frac{(t_{nd} - t_s)}{\mathrm{n}} = -\frac{v_u}{v_p} * \frac{t_s}{n} + t_{pp} \tag{1}$$

This calibration strategy requires measurements at multiple separation times, including the bypass time (t_0), where t_s = 0.01 ms and ions are directed immediately to the time-of-flight mass spectrometer (TOF-MS); the single-pass time (t_1), where t_s = 2 ms and ions complete exactly one circuit of the cyclic separator; and additional user-defined separation times, where the number of passes typically increases with increasing t_s.

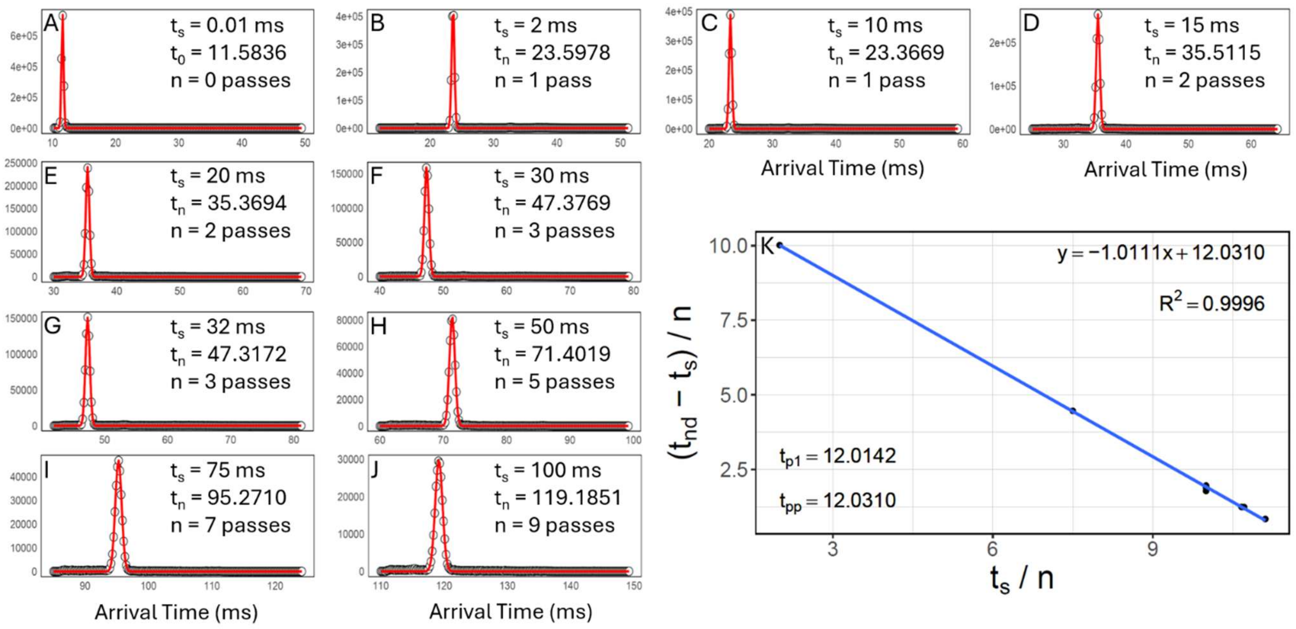

Figure S1. Arrival time distributions (ATDs) for the $[M + H]^+$ ion of the Major Mix standard Val-Tyr-Val recorded at ten different separation times: t_s = 0, 2, 10, 15, 20, 30, 32, 50, 75, and 100 ms (panels A–J). Red curves represent Gaussian fits to each ATD feature. (K) $\frac{(t_{nd}-t_s)}{\mathrm{n}}$ versus $\frac{t_s}{n}$ plot constructed from the Gaussian peak centroids and corresponding calculated pass numbers, used to determine the perturbation-corrected periodic drift time.

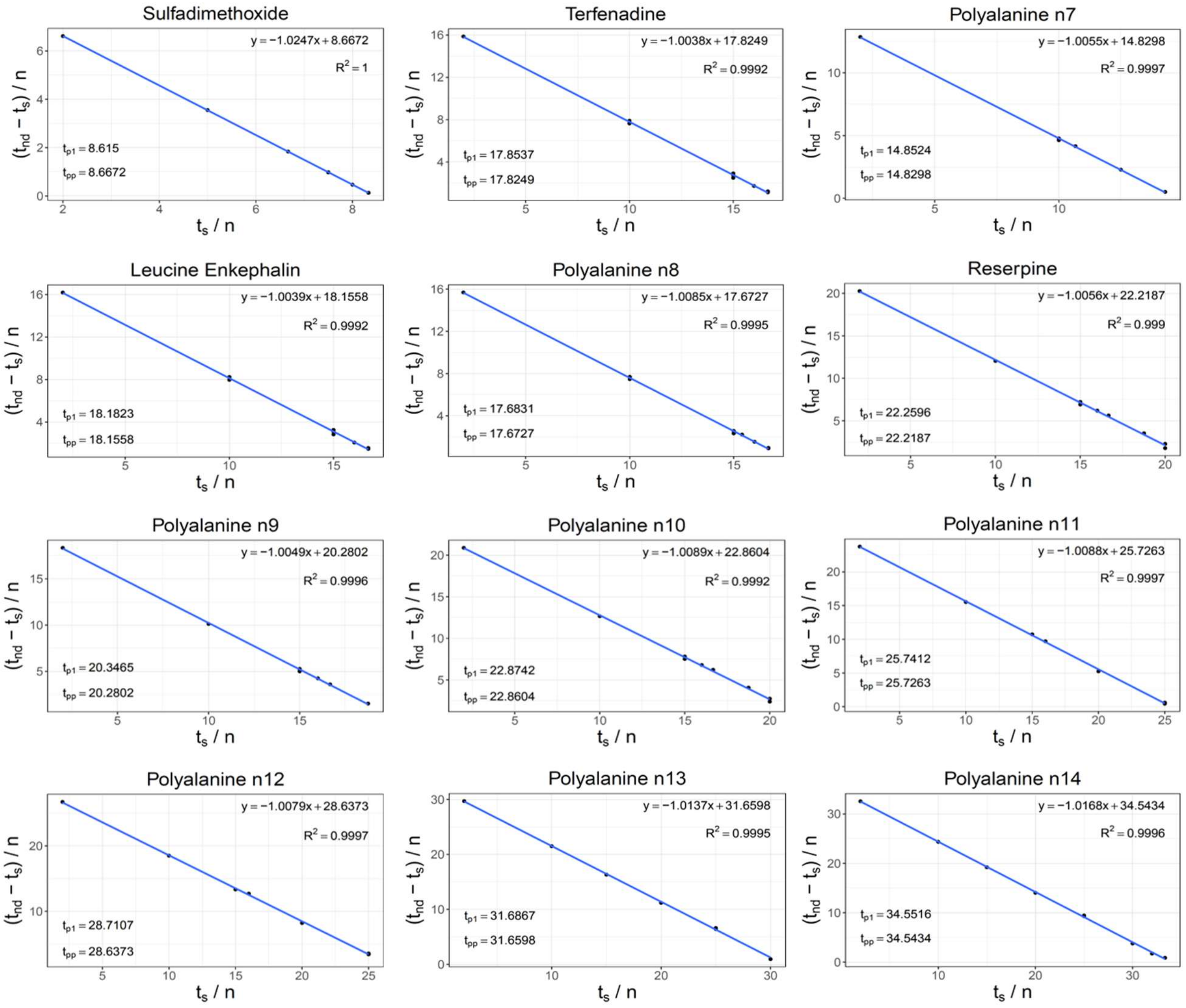

Figure S2. $\frac{(t_{nd}-t_s)}{\text{n}}$ versus $\frac{t_s}{n}$ plots for twelve features in the Major Mix calibration solution. Linear models, corresponding R^2 values, and calculated single-pass and perturbation-corrected periodic drift times are shown for each feature.

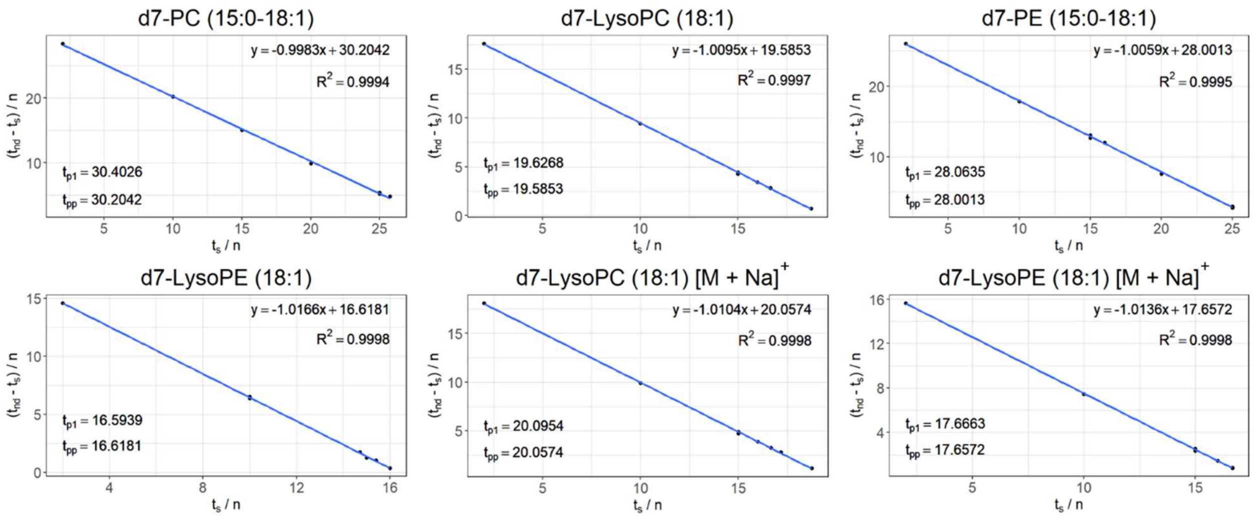

Figure S3. $\frac{(t_{nd}-t_s)}{\mathrm{n}}$ versus $\frac{t_s}{n}$ plots for seven features in the SPLASH II Lipidomix solution. Linear models, corresponding R^2 values, and calculated single-pass and perturbation-corrected periodic drift times are shown for each feature.

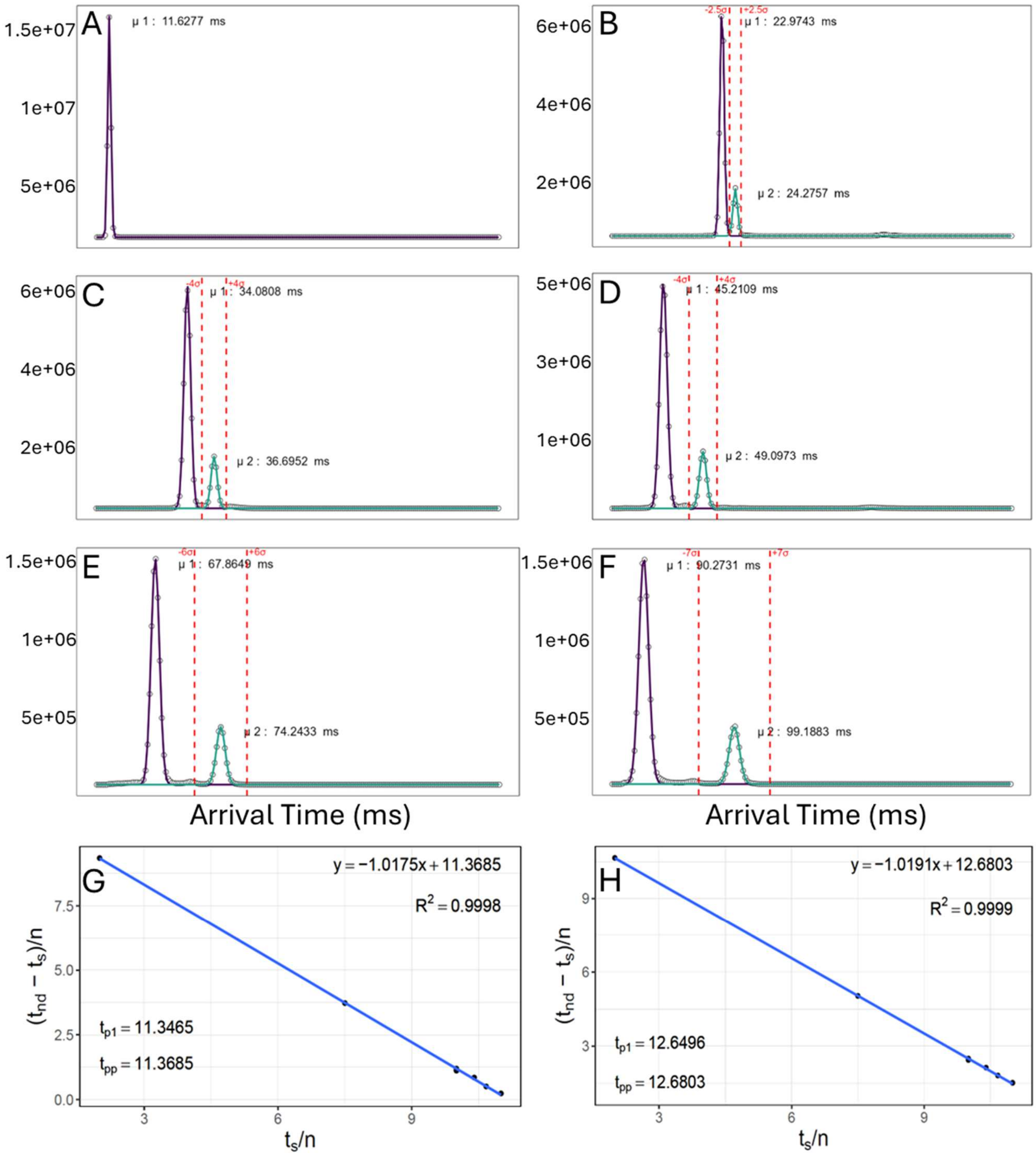

Figure S4. ATDs of the $[M + Na]^+$ ion adducts of prostaglandin isomers PGJ2 in purple and PGA2 in green from 1 to 6 passes in the separation cell (A-F). $\frac{(t_{nd}-t_s)}{\mathrm{n}}$ versus $\frac{t_s}{n}$ plots for PGJ2 (G) and PGA2 (H) created from separated ATDs. Linear models, corresponding R^2 values, and calculated single-pass and perturbation-corrected periodic drift times are shown for each feature.
